# Managing hundreds of improvement teams

**DOI:** 10.12688/f1000research.16099.1

**Published:** 2018-10-31

**Authors:** M Rashad Massoud, Leighann E. Kimble, Victor Boguslavsky, Maina Boucar, Jorge Hermida, Donna Jacobs, Esther Karamagi, Nigel Livesley, Mirwais Rahimzai

**Affiliations:** 1 University Research Co., LLC, Chevy Chase, Maryland, 20815-3594, USA; 2 USAID Applying Science to Strengthen and Improve Systems (ASSIST) Project, Chevy Chase, Maryland, 20815-3594, USA

**Keywords:** Leadership, quality improvement, improvement, teams

## Abstract

Recognizing the notable scale of USAID Applying Science to Strengthen and Improve Systems (ASSIST) Project activities and sizable number of improvement teams, which in some cases is close to 1,000 improvement teams managed in one country at a point in time, we sought to answer the questions: How do we manage hundreds of improvement teams in one country alone? How do we manage more than 4,000 improvement teams globally? The leaders of our improvement programs manage such efforts as though they are second-nature, without pointing to the specific skills and strategies needed to manage thousands of teams. This paper was developed to capture the lessons, considerations, and insights shared in discussions with leaders on the USAID ASSIST Project, including country Chiefs of Party and Regional Directors. More specifically, this paper seeks to describe what is involved in scaling up and managing large numbers of improvement teams. Through focus group discussions and individual interviews, participants discussed the key skills, strategies, and lessons needed to successfully manage large numbers of teams on the USAID ASSIST Project. We concluded that the six key components in managing large numbers of teams are 1) leadership; 2) management structures and capacities; 3) clear and open communication; 4) shared learning, collaboration, and support; 5) ownership, engagement, and empowerment; and 6) partnerships. We further analyzed these six components as being interrelated to one another based on the relationship between culture, strategy, and technique in implementing quality improvement activities.

## Background

**Figure 1.**
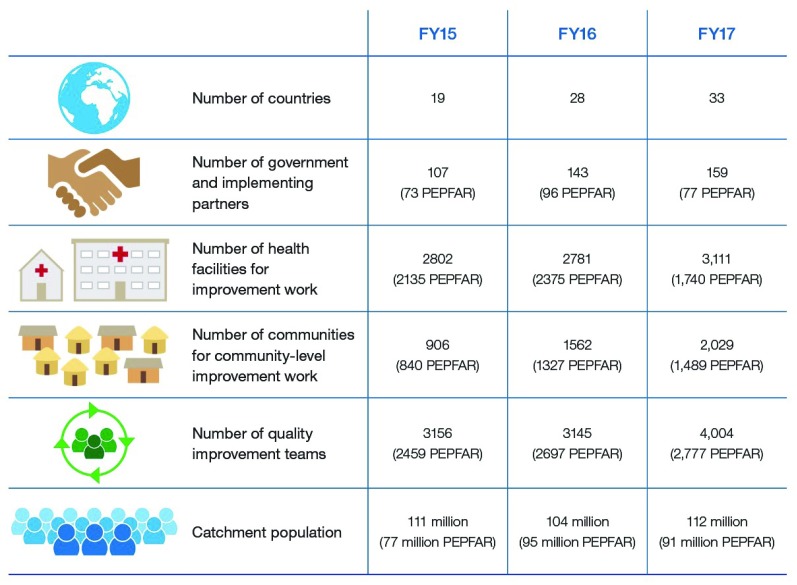
Scale of USAID ASSIST Project.

The USAID Applying Science to Strengthen and Improve Systems (ASSIST) Project is a USAID-funded global mechanism for improving healthcare in low- and middle-income countries. The work under the USAID ASSIST Project is the continuation of the efforts of predecessor projects, USAID Health Care Improvement (HCI) and Quality Assurance Project (QAP) I-III. Over the life of the project, the USAID ASSIST Project was working in 3,111 facilities and 2,006 communities, supporting 4,004 quality improvement teams working in these facilities and communities ( Figure 1). These numbers include both facilities and communities that received both direct and indirect support from USAID ASSIST. Some of the teams were shared between the facilities and communities. In addition to the improvement teams working in the facilities and communities, the ASSIST Project worked with 159 government and implementing partners to improve health outcomes and strengthen health systems globally. Government partners included country Ministries of Health, Ministries of Social Affairs, Ministries of Gender, National AIDS and HIV Control Programs, and other governmental and parastatal institutions. In many countries, our partnerships also included district and provincial health management teams and leaders.

The estimated total number people served by the health facilities benefitting from the efforts of these QI teams was 112 million people. USAID ASSIST works with host country national and local level counterparts in providing technical assistance to meet their aims for improving healthcare outcomes in priority areas. This work is done by forming teams among the service delivery providers and managers in counterpart institutions to work on improving the quality of care delivered to patients. Over the life of the USAID ASSIST Project, USAID Country Missions and USAID Offices (such as Health Systems, HIV/AIDS, and Maternal, Child, Health, and Nutrition) have played an instrumental role in facilitating our ability to collaborate and coordinate with partners to expand the reach of our work.

In Uganda alone, at the end of June 2017, ASSIST was working with 928 quality improvement teams to strengthen the health system; prevent HIV; improve care and treatment for people with HIV; and improve family health related to maternal, newborn, and child health, family planning, nutrition, tuberculosis, and malaria. At the same time, in Tanzania, ASSIST was working with 781 quality improvement teams, including spread and scale-up of preventing mother-to-child transmission and antiretroviral therapy activities. Working with many teams is not unfamiliar to the USAID ASSIST Project. ASSIST and its predecessor projects, HCI and QAP, have supported thousands of different improvement teams to accomplish their improvement aims. The USAID series of quality improvement contract mechanisms have been at the heart of a global movement in taking successful improvements to scale ^1, 2^. It became evident that successful spread depends on the adaptation of key principles to context and using “hybrid models” based on lessons learned from successful scale-up efforts ^3^.

The progression between the predecessor projects and the work on ASSIST today reflects the impact of successful scale-up of quality improvement. Starting with QAP II’s initial large-scale spread efforts in Tula and Tver in the Russian Federation ^4^, the foundations for large-scale spread efforts were developed. These efforts were continually evolved over QAP III, HCI, and ASSIST. Today this is particularly the case in India, Uganda, and Tanzania, which each started off with a small number of teams. The success of improvement at a small scale in each of these countries gradually spread to other health centers and districts throughout the country, leading to a larger number of improvement teams able to reach a larger catchment area.

A brief overview of the work in each of these countries as well as a summary on spread can be found in **Supplementary File 1**. More information can be found in the FY17 Country Reports for each country, as well as on the USAID ASSIST website ( www.usaidassist.org).

## Introduction

Beyond the use of improvement methodology and spread methods in country activities, there are several interrelated actions and communications that must take place to not only manage current improvement activities, but to scale up improvement activities that are yielding positive results ^5^. This paper was developed to capture the lessons, considerations, and insights shared in discussions with leaders on the USAID ASSIST Project, including country Chiefs of Party and Regional Directors. More specifically, this paper seeks to describe what is involved in scaling up and managing large numbers of teams ^6, 7^.

Recognizing the notable scale of USAID ASSIST Project activities and sizable number of improvement teams, with in some cases close to 1,000 improvement teams at a point in time, we sought to answer the questions: How do we manage 100s of improvement teams in one country alone? How do we manage more than 4,000 improvement teams globally? The leaders of our improvement programs manage such efforts as though they are second-nature, without pointing to the specific skills and strategies needed to manage thousands of teams.

## Methods

### Study design

The underlying objective for this research began with a question from the USAID ASSIST Project Agreement Officer’s Representative (AOR) in a USAID ASSIST Quarterly Review Meeting (QRM). In response to a report on the large number of improvement teams in Tanzania and Uganda on the USAID ASSIST Project, the AOR asked the question, “how do you manage 1000 teams?”.

Because this topic is specific to the work on the USAID ASSIST Project and involves a small number of individuals, there was no formal sampling method involved. The findings in this work are from the Project Director, Senior Improvement Advisors, Regional Directors, and Chiefs of Party that were available and willing to participate in the focus group discussion via Zoom videoconferencing, one-on-one interviews, and email discussions about the topic of managing large numbers of teams.

The insights analyzed in this paper were obtained through a series of discussions with the participants. Each of these individuals has many years of experience using quality improvement to yield improved health outcomes in low- and middle-income countries. The group formed encompasses all Regional Directors, Chiefs of Party, and Senior Improvement Advisors available to join the Zoom videoconference and that had experience managing hundreds of teams on the ASSIST Project, in addition to the Project Director. As the experiences are specific to the ASSIST Project, only participants with experience managing on the ASSIST Project were selected for participation in the group discussions and individual interviews. Initial discussions occurred over Zoom videoconferencing meetings and included the authors listed for this paper. The method of data collection began with the use of a larger focus group of participants to explore the topic of how the USAID ASSIST Project manages teams, including lessons learned from the experiences of the participants. The discussion was semi-structured, with the researcher asking questions only to probe deeper discussion or to obtain clarification. The opening question for the focus group stemmed from a previous USAID ASSIST QRM and was the broad question “How do we manage 1000 teams?”. Participants in the focus group and in the interviews and email discussion has the background for this question from attending the USAID ASSIST QRM.

Notes were taken by interviewer throughout these calls, and insights from the meetings documented. The meeting minutes, which were initially a series of quotes from the meeting participants, were then circulated after the group meetings for additional feedback, ideas, and input regarding experiences managing improvement teams.

Additional one-on-one calls and in-person meetings were held following the group virtual conversations over Zoom to gain more detail from individual participants about what they found important in successfully managing teams in their experience on the USAID ASSIST Project, as well as in their broader experiences working in quality improvement. The input from these one-on-one meetings allowed the interviewer to get a more in-depth account of individual experiences based on the individual’s technical area of expertise, number of years of experience, and country/regional experience in improvement.

The interviewer for the focus group and individual interviews (LK) is an Improvement Advisor with University Research Co., LLC and the USAID ASSIST Project. Leighann holds a Master of Arts in International Relations and has worked with University Research Co., LLC and the USAID ASSIST Project for over four years. Her credentials and experience working with the USAID ASSIST Project provided her with the background needed to probe discussion and interview questions related to the work of the project. LK was also familiar with the participants, having had several interactions over the years with each of the participants. She has not directly managed large numbers of teams in the field, which allowed her to be objective in asking discussion and interview questions without interjecting her bias, thoughts, or experiences into the conversations with participants.

### Data analysis

Data from the focus group discussion was initially recorded in the form of quotes from the participants, thereby transcribing the conversation. The interviewer, along with the Project Director, then found common themes between the quotes and categorized the quotes. The updated and categorized list of quotes was then circulated to the participant group to receive feedback, request clarification, and confirm that quotes were placed in the appropriate categories. Several iterations of this process were completed to revise, expand, and clarify each of the categories, while circulating the revisions for group feedback.

The feedback from the one-on-one meetings was combined with the input from the group discussions to find common themes and lessons learned from conversations on how to manage hundreds of teams. The synthesized ideas were circulated to all participants in the initial group discussions for additional feedback, clarification, and revisions. In the analysis to follow, quotes open each subsection, reflecting direct quotes from the conversations held with USAID ASSIST staff described above. The analysis is derived from a synthesis of inputs, feedback, several rounds of group discussion, one-on-one interviews, and rounds of revision to generate key ideas and lessons learned on how the USAID ASSIST Project has learned to manage hundreds of teams.

Once the categories were finalized, data analysis involved extracting the meaning behind the quotes in each of the categories and identifying the key lessons in each of the six categories as applied in USAID ASSIST Project activities. With feedback from the Project Director and further feedback from the larger group, the categories were further analyzed based on the theoretical framework of the Quality Management Triangle. Analysis was conducted based on the Quality Management Triangle to draw more depth to the results and conclusions of the discussions, particularly as they relate to the content of the six interrelated categories. Once finalized, the draft version of the paper was circulated to a smaller group on the USAID ASSIST Project for review and several rounds of revisions, prior to sending to USAID for further review and approval.

## Analysis

### The quality management triangle

**Figure 2.**
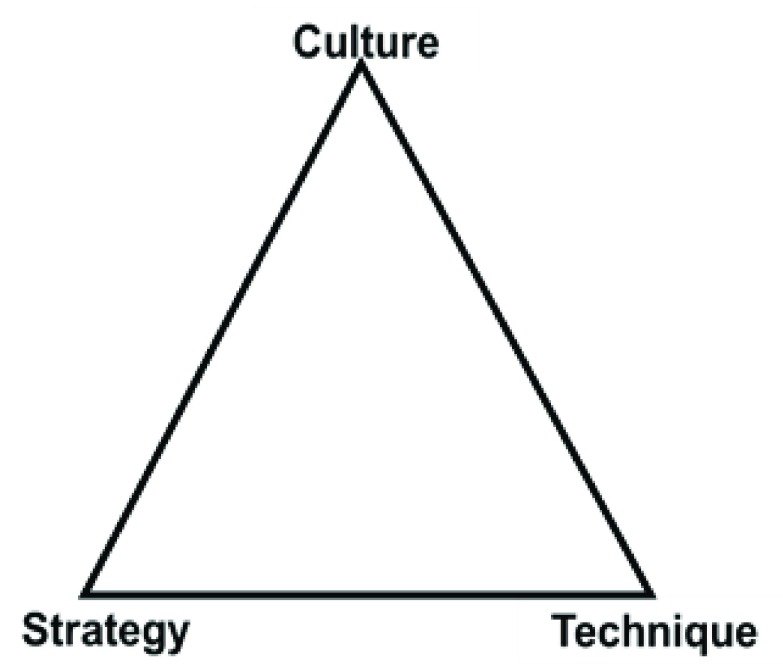
Quality management triangle.

As depicted in Figure 2, there are three central components for effective quality management: 1) a culture of managing for quality; 2) improvement technique; and 3) strategy for implementation. To effectively lead healthcare quality improvement, the culture, technique, and strategy for implementation must be complementary and adapted in consideration of the context. We highlight the interaction between culture, strategy, and technique due to the overlap between these three factors in quality improvement and in scaling up. As will be reflected throughout the paper, understanding the overlap between these factors and addressing each factor is important because the *technique* of quality improvement is only successful when applied using the appropriate *strategy* in the given *culture*.

Quality improvement requires continual adaptation to context, which is facilitated through constant coordination, review, and feedback, each managed through six interrelated categories of action. When talking about improvement, people most often refer to the technique of applying quality improvement methods to achieve improved health outcomes. The technique encompasses testing and implementing changes to processes of care in a health system to yield improved outcomes. However, as depicted in Figure 2, the technique of quality improvement alone is not sufficient – the culture and strategy surrounding the technique are essential to achieve results. Culture refers to both internal and external factors (i.e., management structures, teamwork, communication environment, etc.) that may support or inhibit quality improvement in the given context. Changes may need to be made to culture to create an environment conducive to quality improvement. Strategy involves the actions needed to meet external needs, in this case improving global health priorities, while making changes to internal processes of care to yield improved outcomes.

Success in quality improvement relies on integration within the culture, strategy, and technique specific to the context in which the improvement activity is taking place. This paper provides an overview of how each of these categories has become characteristic of our work, providing examples from activities throughout the USAID ASSIST Project.

### Six key components for managing hundreds of improvement teams

Through discussion with our project leaders in country as well as at headquarters, we distilled the key components to managing our work successfully. These components fall into six interrelated categories:

1. Leadership
2. Management structures and capacities
3. Clear and open communication
4. Shared learning, collaboration, and support
5. Ownership, engagement, and empowerment
6. Partnerships

The connection between these six categories can be understood within the relationship between culture, strategy, and technique in quality improvement, as will be further described below.

***Leadership***

*“Technical capability is not enough; leadership is needed [for cultural transformation].”*

As highlighted in Table 1, leadership at all levels is key in managing and supporting improvement teams. Local leaders are the individuals that live and work in the communities and facilities in which changes are to be made to yield improvement. The role of the local leader is to support the improvement team in making changes to processes of care and problem-solve any issues that teams cannot fix at their level. Leaders at the district and national levels provide ongoing support and problem-solving beyond the boundaries of the specific facility or community. When multiple local teams are working on the same improvement activity in a district, the role of the district leader becomes more than just supportive – the district leader plays a key role in facilitating collaboration and knowledge sharing between the improvement teams within the district. Likewise, if the improvement team is happening on a national scale, the role of national leadership grows into one that supports collaboration and knowledge sharing between teams across multiple districts.

**Table 1.** Insights about leadership.

	Leadership
**Culture**	- Appreciation of staff and their efforts - Leadership at all levels (beginning with entry-level staff all the way to the top) - Recognition of leadership at all levels ° Requires a shift in power ° Deliberate recognition of capabilities of individuals at all levels, not just at the top - Identifying leaders and potential leaders on QI teams - Creating “champions” - Leadership and decision making as not just top-down. Must engage all levels in work - Capable of defining and celebrating excellence/success
**Strategy**	- Ability of leaders to identify what the priorities are for improvement and involve individuals to contribute to the improvement to the limit they can (providing them with the opportunity to be involved and grow) without setting them up for failure - Leading the team
**Technique**	- Convening, empowering, engaging staff, problem-solving - Role-playing - Mentorship (by good leaders) that guide, encourage, and create opportunities for new potential leaders

While ASSIST provides technical assistance in improvement, we recognize that the work of our improvement teams goes beyond building technical capability – leaders must have the capacity to lead improvement and must be engaged and committed to use their capabilities. Within our work, we find that supporting local leadership by creating both the capability and capacity for leading improvement is essential. On one hand, capability involves having the clinical and improvement knowledge and skill needed to improve healthcare results. However, capability on its own is not enough to translate into action. Leaders must be able to use these capabilities to lead improvement. Capacity involves leaders having the empowerment and confidence needed to apply their knowledge and make changes to the processes of care in their health system to yield better results.

Much of ASSIST’s work in leadership includes engaging leaders in host countries to support the work of the improvement teams and providing them with the knowledge and skills to do so. For example, we encourage leaders to allocate time and resources for teams to meet, participate in reviews of data, attend and present at learning sessions, chair non-ASSIST supported technical meetings where results were presented, facilitate decisions to institutionalize teams’ efforts, present results at regional, national or international meetings, etc. Our understanding of the key roles of leadership for leading improvement teams is highlighted in Box 1.

**Box 1. Key tasks for leading improvement teams**

1. Set clear goals for all staff.
2. Give all staff the freedom and confidence to figure out how to do their work. Avoid micromanaging.
3. Be available to help with problem solving if things are not going well. Help staff learn from their mistakes rather than blaming and shaming.
4. Focus on the positives and actively look for success and new opportunities.
5. Celebrate the successes and positives.
6. Identify and address failures as system failures, and not individual failures.

By modeling and encouraging a “culture” that empowers and supports local leadership, ASSIST provides technical assistance to local leaders at various levels of the health system to build their confidence, empowerment, resources, and tools to help them lead local improvement teams. In addition to providing technical assistance related to capacity, or knowledge and “know-how”, for improvement, the resources we provide to leaders include problem-solving skills and ability to develop team dynamics to support improvement. With these skills and knowledge, we give local leaders the sense of accountability for leading improvement teams and reaching improvement aims and goals set by the team. While the overall strategy of quality improvement is to embed quality within the system, the “technique” involves using quality improvement to develop the capacity of individuals to lead the work over and above individual facilities. This too requires engagement and open communication with leaders to ensure they are prepared, capable, and empowered to lead quality improvement. The “strategy” of developing leadership involves providing local leaders the technical assistance they need to be technically capable. Beyond this is the component of empowerment and support of individuals to be capable as leaders accountable and empowered to sustain improvement independent of the project.

***Management structures and capacities***

*“Most of the successful events that we have had built on prior successes and utilize champions within it (whether or staff or in MOH). [This] requires meticulous management and does not happen without very serious attention to detail and a strong pattern of engagement.”*

For an activity to operate properly, there must be a management structure. In the case of improvement activities, because of the complexity and engagement needed, the “technique” required is the ability to develop structures to support individual quality improvement teams and to allow people to convene and coordinate—not only at a local level but on national, regional, and global levels— and structures to oversee all elements of the work and adapt it as necessary. The USAID ASSIST Project works with existing management, government, and organizational structures, adapting to the context of these existing structures to yield improved results in health system processes.

Management structures can be facilitated by creating roles for coordinators, points of contact, etc., that are appropriate for the context. For instance, in developing roles, one improvement coach should be identified for every 5–15 facilities, with one supervisor for every 5–10 improvement coaches, and one coordinator for every 5–10 supervisors. These proposed numbers come from many years of experience; too many improvement coaches and supervisors create over-saturation in the facilities, while too few makes it difficult to manage the teams. Improvement coaches convene the improvement teams, supports leaders, and actively builds the improvement teams. Supervisors support the improvement coaches by accompanying improvement coaches when convening improvement teams to provide additional guidance and monitor the progress of the improvement teams and the work of the improvement coaches. Coordinators provide overall oversight of supervisors to ensure consistency across the improvement team activities. The strategy for maintaining a management structure requires accountability and continuity, with attention to detail.

Accountability occurs at multiple levels, with districts holding facilities accountable for improvement, supervisors and coaches holding staff accountable, and staff holding themselves accountable to improvement activities to support the work and progress of their improvement team. In managing improvement activities, improvement teams must have the structure to convene regularly in combination with the accountability to take action in between meetings to make the changes needed for improvement and collect data to keep track of the results related to those changes.

The culture of a successful management structure requires communication, meticulous planning, and engagement ( Table 2). Meticulous planning requires attention to detail in managing teams. Such planning requires specific schedules of activities with clear deliverables, follow-up, timelines, and individuals assigned to keep meetings and activities on track. In the process, schedules and activities must be monitored and adjusted based on reality to make the schedules “living” rather than “static”.

**Table 2.** Insights about management structures and capacities.

	Management structures and capacities
**Culture**	- Develop leaders at all levels with the capacity to carry out their roles - Must have access to management for problem-solving and managers with the capacity to problem-solve - Promote teamwork and clear and open communication
**Strategy**	- Set up appropriate teams with the right leadership to tackle priority areas - Authorize and empower teams to test and implement changes
**Technique**	- Establish review meetings to problem-solve and support teams - Establish structures for communication (meetings, team development)

Leadership engagement in activities must involve key members of leadership, improvement coaches, frontline staff delivering healthcare services, and administration, each communicating with one another to work towards a common goal. Communication, as will be described further, should be open, honest, and free of blame. At the heart of management structures lie individuals that can be leveraged as “champions” in supporting and maintaining the structure.

***Clear and open communication***

*“Communicating with teams on a regular basis to gather learning and push this information back out to the other teams.”*
*“Meet with key people at national level, local level, and district level to let them know what the project is about and what it can do and what they can do to support it.”*

Just as structures are important, communication with individuals and improvement teams within these structures is essential. Communication allows improvement teams to learn from one another and to ensure that teams have a clear understanding of what and how they are going to improve. Interaction with people at the national, local, and district levels allows the USAID ASSIST Project to articulate what our project is about and how we can work with USAID Missions, Ministries of Health, local entities, and partners to improve healthcare. Our lessons for clear and open communication are detailed in Table 3 and Box 2.

**Table 3.** Insights about clear and open communication.

	Clear and open communication
**Culture**	- Definitions of failure and success are known - No fear of expressing or communicating failures (no fear of failing) - Setting expectations for the work (improvement) and how to communicate regularly about developments (or lack of) in the work - Improvement as a never-ending journey
**Strategy**	- Make channels of communication known - Communication is multi-way, not one-way - Things communicated are acknowledged, reviews, and acted upon, as necessary
**Technique**	- Develop a means of communication between teams - Feedback mechanisms - Create means of communication between leadership and staff

**Box 2. Key Lessons for clear and open communication**

1. Use internal communication within the project to actively problem-solve and share successes.
2. Identify and discuss solutions for common challenges.
3. Communicate challenges that need support to solve (i.e. internal sharing meetings, internal quality improvement skills-building meetings, case studies, ad hoc meetings, etc.)
4. Develop mechanisms to share learning between facilities and between people managing the program both within ASSIST and in government.

Different country contexts involve different scenarios, including: countries where such structures and communication schemes are in place at all levels; countries where efforts are mostly at decentralized levels without formal structures at higher levels; and countries in which there are horizontal approaches in disease and health programs and where such structures ally specific management bodies. Recognizing there are different improvement approaches and terminology, regular communication also helps to avoid confusion or misunderstanding. Clear and open communication includes the communication between ASSIST and improvement teams at learning sessions; communication between coaches and teams during coaching visits; and communication between these sessions. For areas in which internet access was available, communication occurred through emails, WhatsApp and other mobile applications, SMS messaging, and Zoom videoconferencing, as available.

Communication must occur on a regular basis. Improvement teams keep ongoing record of their work, reporting on this work to their supervisors and to the USAID ASSIST Project. Regularity of interaction encourages improvement teams to problem-solve, learn from improvement progress and challenges, and recognize steps for sustaining and institutionalizing improvement. As will be discussed in the next section, a key to improvement activities is learning. Learning requires open communication between individuals and improvement teams. Open communication means that the culture allows for communication of both successes and failures without fear of blame or penalty. Clear and open communication also assists in setting the stage for shared learning, collaboration, and support in our work, as will be discussed below.

***Shared learning, collaboration, and support***

*“Connecting people so that one hospital [or health center] can help another.”*

Management structures must allow for clear and open communication by accepting and acknowledging both failures and successes as learning opportunities. Developing such a management structure requires management actions that support and create opportunities for open communication, collaboration, and engagement of staff. By encouraging staff to communicate and work together, a culture of shared learning and collaboration can be supported. This communication also provides key feedback to management on what is working and what is not, to continually fine-tune strategy and technique.

Communication is transferred into shared learning, collaboration, and support not only horizontally across health facilities, but vertically between leadership and indirectly with partners. For instance, health facilities may need to communicate and collaborate vertically with local leaders to solve ongoing problems within the facilities, or vice versa. Similarly, shared learning, collaboration, and support occurs when we work with partners with specific strengths and expertise to solve specific problems. In this relationship, we also offer our expertise and resources to support, collaborate with, and problem-solve with our partners and other local entities. Our approaches and lessons for shared learning, collaboration, and support are illustrated in Table 4.

**Table 4.** Insights about shared learning, collaboration, and support.

	Shared learning, collaboration, and support
**Culture**	- Leadership is engaged in the learning to learn from what their teams and others are doing - Judgement-free zone that focuses on mutual learning and improvement
**Strategy**	- Learn from the successes and failures of other groups - Assist one another in achieving goals and overcoming obstacles - Recognizing commonalities that allow for future learning - Continuous improvement as a strategic approach
**Technique**	- Develop communication and structures for shared learning and continuous improvement

For instance, using the “technique” of making connections, improvement teams can not only share their experiences with one another, but learn from their successes and failures. Because teamwork is necessary for improvement, shared learning and collaboration sets a tone and “strategy” for scale-up planning and creates a format for people to work together for larger-scale outcomes. This is the “culture” of attention to detail required for such planning and coordination. Through shared experiences, people can come together and work towards a common goal, learning from their experiences along the way.

***Ownership, engagement, and empowerment***

*“Allowing [improvement] work to be given to local authorities so that it can be taken over from us. We need to lead, motivate and encourage them. It is also important to bring them together.”*

In addition to leadership, management structures, and a focus on learning, the sustainability of activities under the USAID ASSIST Project is dependent on creating a “culture” in which local leaders and individuals are engaged and have a sense of ownership and empowerment to continue the work. Engagement requires ongoing negotiations and conversations with the Ministry of Health, local government and authorities, and other stakeholders in the health sector. Conversation itself is not enough. Local entities must feel invested in these activities and must feel encouraged to continue the work.

When the USAID ASSIST Project engages in work with a country, initial discussions emphasize that we are in the country to help and describe what the role of ASSIST is and how frontline workers can do their work and collect data so that we may support their improvement activities. We also make sure to engage in conversation with municipalities and districts as their leadership, support, and engagement in the work within their region is necessary for accountability to trickle down to the facility and community levels. The Chiefs of Party for the USAID ASSIST Project play a key role in communicating with the Ministry of Health to create and maintain ownership and accountability. The lessons from our work in increasing ownership, engagement, and empowerment of local actors under the USAID ASSIST Project is highlighted in Table 5. Practical approaches for engagement are described in Box 3.

**Table 5.** Insights about ownership, engagement, and empowerment of local actors.

	Ownership, engagement, and empowerment of local actors
**Culture**	- Ownership, engagement, and empowerment at all levels, working together with local actors (healthcare providers, families, communities) - Setting a standard for responsibility to improvement and ownership of results - Creating an environment that celebrates excellence and communicates failures to learn from them (this requires clear and open communication) - Set a model/example of desired actions and behaviors ° Accountability to results and pride in one’s work ° Recognizing one’s role and effect on results
**Strategy**	- Use empowerment and accountability as means of scaling up and spreading improvement - Use improved results yielded by testing and implementing changes to develop new standard operating procedures; this creates empowerment by creating a new and improved standard - Give local actors the ability to change the way in which things happen (test and implement changes)
**Technique**	- Have local actors map existing processes and documents the changes they are making to them to get better results - Once local actors have achieved their results, they create new process maps which become the new standard. They then work with others (departments, units, teams, etc.) to spread and scale results/improvement to yield better outcomes of care in other areas - Structure effective delegation of the task of managing the demonstration and spread effort to the appropriate leaders in the system

**Box 3. Four approaches for engagement**

1. Focus on results that matter to the people who will own the work.
2. Link people new to quality improvement with influential people or groups that have successfully used quality improvement approaches.
3. Support people with good results to talk widely about their results and help them show their results to important figures.
4. Encourage people to talk about the quality improvement methods that they use to achieve good results.

Ownership, engagement and empowerment must exist not only with local government and authorities but must be rooted within improvement teams. Staff in facilities must have ownership over their work and be engaged with the work they are doing in their facility. Leaders within facilities, whether supervisors or coordinators, then engage and empower the staff they are working with, help them achieve their goals, and help them remain accountable to their work. One way that ASSIST encourages ownership, engagement, and empowerment in facility staff is by helping them get results quickly and promoting these results. Achieving positive results requires us to help new quality improvement teams choose aims that are more likely to be attainable. Initial aims must therefore:

1. Be easy to measure without creating new measurement systems
2. Be related to processes that are largely under the control of the improvement team
3. Involve processes that occur frequently so that data can be measured more frequently and changes are more visible (issues of routine care are better than issues of complications management, for instance)
4. Benefit the improvement team if the processes are improved (i.e., the team’s supervisor will be happy with the results, and patients will be happy with the results)

The “technique” of quality improvement requires multidisciplinary teams and the involvement of individuals at all levels of healthcare delivery. The focus on improvement engages not only “experts” in improvement but empowers others to play a role in quality improvement, engaging them as active members on a team working towards improved health outcomes. By engaging the Ministry of Health, implementing partners, and other local entities into our work, a “strategy” is created in which improvement is built into the system. Engagement of the Ministry of Health should begin at the start of the activity, keeping up-to-date visits and contact with officials. Through this communication, support of the Ministry of Health should be requested and successes celebrated throughout the course of the activity. At the end of the activity, support for the work is to be handed over to the Ministry of Health.

In some health systems, countries have developed national policy and strategies on quality improvement that have integrated roles and responsibilities for quality in the jobs of staff at all levels. In other instances, countries may wish to develop gradual plans to operationalize quality improvement into their health system. By actively working with government counterparts at local, national, and regional levels, we can ensure that they are engaged in a way that fits improvement into their own structures.

***Partnerships***

*“Partners are working together with the team, maintaining communication to facilitate shared learning and creating tools that allow teams at the local level [to use them].”*

The role of partners in our work allows us to broaden the range and scope of our work in improving healthcare. Our partners include both government and non-government entities, with host country national (HCN) partners being local organizations. In July 2017, ASSIST worked with 159 government and implementing partners in improving health outcomes. In the case of our partnerships, our “culture” is one of a shared vision and collaboration. Because of this culture, our work in some countries, such as in India, the support of the improvement work of the USAID ASSIST Project occurred primarily through partners. In our work with implementing partners (IPs), the USAID Mission or office that provides funding may decide whether we are working with implementing partners through joint planning of activities, coordination, progress review, reporting, etc. In the cases in which we work with implementing partners, the leadership of USAID leads the work and directs the partnerships. While some of these agreements are formal agreements with signed Memoranda of Understanding between parties or an established partnership established by USAID, many others are informal agreements developed through a relationship built through ongoing collaboration and coordination with the partner entity.

By working in collaboration with partners, we engage and maintain communication with a larger number of improvement teams, who can share their expertise and experiences so that teams are learning from one another and sharing tools that can improve their work. The support of our partners in the form of technical and human resource support also allows us to make local support available and accessible to our teams locally through universities and local experts. Our lessons in working with partners are detailed in Table 6.

**Table 6.** Insights about partnerships.

	Partnerships
**Culture**	- Collaborative and working towards a common goal - Includes local organizations, government, private sector, local leaders, experts, communities - Work with and through implementing partners (IPs; when available)
**Strategy**	- All partners are actively involved - Government partners/system partners take the lead spread through their regular mechanisms (meetings, events, etc.) - We work with IPs and enable them to do our work in the spread phase
**Technique**	- Nurture capability of system partners to improve, including leadership and spread from Day 1 - Adapt spread needs to existing ways of operating their system - Enable IPs (when available) and government partners to coach local trams to be able to reach a larger number of teams than we would be able to reach alone

Meeting Notes This data is de-identified. Click here for additional data file. Copyright: © 2018 Massoud MR et al. 2018 Data associated with the article are available under the terms of the Creative Commons Zero "No rights reserved" data waiver (CC0 1.0 Public domain dedication).

## Conclusion

The work of the USAID ASSIST Project relies on the success of quality improvement teams worldwide. Improvement teams must have both the technical and leadership capability to carry out and sustain improvements to improve healthcare outcomes. To manage these improvement teams, we have developed a management structure with a “culture” of quality, engagement, and empowerment. With the “technique” of quality improvement methodology at the center, we have used the “culture” of clear, open communication and shared learning and collaboration. By connecting improvement teams, teams can share information and lessons learned from their successes and mistakes, sharing these experiences with one another in a way that promotes learning and teamwork to reach a larger scale. The work of the USAID ASSIST Project on such a large scale and across more than 4,000 improvement teams globally would not be possible without the involvement and engagement of local leaders and partners, who share a vision and dedication to improving healthcare outcomes and strengthening healthcare systems.

## Ethical statement

The involvement of all meeting participants and those involved in email correspondence for this study was entirely voluntary. Participants involved in meeting discussions, interviews, and email correspondence were made aware that the discussions would be documented for use in this study. Participants were made aware that their participation in the discussions and email correspondence used for this study were voluntary and that they could choose to no longer participate at any time.

## Data availability

The data referenced by this article are under copyright with the following copyright statement: Copyright: © 2018 Massoud MR et al.

Data associated with the article are available under the terms of the Creative Commons Zero "No rights reserved" data waiver (CC0 1.0 Public domain dedication). 

The data provided for this study is in the form of meeting notes ( Dataset 1) and email correspondence with participants. Since the email correspondence may contain potentially identifying information, this is not openly available. Those interested in accessing this data may contact Leighann Kimble at lkimble@urc-chs.com with any questions or requests. Data will be accessible under the following conditions <insert conditions>.

F1000Research: Dataset 1. Meeting Notes, 10.5256/f1000research.16099.d222292 ^8^

## Footnotes

^1^World Health Organization (WHO). An Approach to Rapid Scale-up: Using HIV/AIDS Treatment and Care as an Example. 2004.

^2^Massoud MR, Nielsen GA, Nolan K, Nolan T, Schall MW, Sevin C. *A Framework for Spread: From Local Improvements to System-Wide Change*. IHI Innovation Series white paper. Cambridge, Massachusetts: Institute for Healthcare Improvement; 2006. (Available on www.IHI.org)

^3^Beracochea E. (Ed). “Scaling Up High-Impact Interventions in Health Care”. *Improving Aid Effectiveness in Global Health*. Ref AID Book ppls P8 AID effectiveness book in a box; Box with approaches P7 AID effectiveness book; Lessons learned box P10 AID effectiveness book; 2015.

^4^Berwick DM. Lessons from developing nations on improving health care. *BMJ* 2004;328: 1124–9.

^5^Langley GJ, Moen RD, Nolan KM, Nolan TW. *The Improvement Guide: A Practical Approach to Enhancing Organizational Performance*. Second Edition. San Francisco, CA: Jossey-Bass; 2009.

^6^Massoud MR and Mensah-Abrampah N (2014) A promising approach to scale up health care improvements in low-and middle-income countries: the Wave-Sequence Spread Approach and the concept of the Slice of a System [v1; ref status: indexed, http://f1000r.es/37o] *F1000Research* 2014, 3:100 (doi: 10.12688/f1000research.3888.1)

^7^Massoud MR and Mensah-Abrampah N. “Scaling-up of High Impact Interventions”. Ed. Beracochea in Aid Effectiveness in Global Health: How to Realize Global Health Goals. Springer 2015.

^8^Massoud MR, Kimble L, Boguslavsky V *et al.*. Dataset 1 in: Managing hundreds of improvement teams: A case study of the USAID Applying Science to Strengthen and Improve Systems Project. *F1000Research* 2018, (doi: 10.5256/f1000research.16099.d222292)

## Supplementary material

Supplementary File 1: ASSIST country examples of managing hundreds of improvement teams.
