# Supplementary material for: Managing hundreds of improvement teams
Source: F1000Res. 2018 Oct 31;7:1722. [Version 1] doi: 10.12688/f1000research.16099.1 (PMC6305208; doi:10.12688/f1000research.16099.1)
Supplement: Meeting Notes — This data is de-identified. [file f1000research-7-17579-s0000.tgz › 3a105b6e-6d7e-4719-aa02-9da225831346_Dataset_1_-_Notes_How_do_we_manage_1000_teams.docx]

How do we manage 1000 teams?

***“Communication, coordination, and structure within the project itself.”***

[Management] structures (for each of the below)

- “there must be a structure that allows people to meet, have a schedule, and convene individuals on a local level”
- Ex. Regional coordinators as first points of contact for facilities to help meet facility needs
- Need for accountability and continuity through management structure
- “To manage at large scale requires the setup of a structure.”
- Support structures are needed….“What do you do when you get off the plane?”…
- Most of the successful events that we have had builds on prior successes and utilizes champions within it (whether or staff or in MOH). Requires meticulous management…. Does not happen without very serious attention to detail and a strong pattern of engagement….
- “Should we be using a more integrated approach through which going to scale needs to be built in? This would be developing a model with strong institutionalization principles”

Clear, Open Communication

- “A big part in working with these teams is communication”
- “Communicating with teams on a regular basis to gather learning and push this information back out to the other teams”
- “Meet with key people at national level, local level, and district level to let them know what the project is about and what it can do.”
- “Need to communicate objectives and plans…”
- “Communication [with partners] is important as well as being explicit regarding improvement approaches and improvement languages.”
- “Also learned how the nature of what we are trying to improve is very critical…”
- “Very important to be sure there is regularity of meetings on the local level and to allow social services to learn from improvement teams about the progresses and challenges and institutionalize… creating a document that supports institutionalization. People need to understand why this is important.”

Shared Learning and Collaboration

- “Connecting people so that one hospital can help another”
- “All [scale ups] required collaboration, which sets the tone for something be bigger.”
- “What are we trying to accomplish and what are the good means and bad means of doing this… and learning from this.”
- Creating a format for people to work together to make sure everyone is on board and why they are working together… sharing the same view on why and what we are doing

Ownership, Engagement and Empowerment:

- “Allowing work to be given to local authorities so that it can be taken over from us. We need to lead, motivate and encourage them. It is also important to bring them together.”
- “Need to work and negotiate with government, other projects and implementing partners working in the existing area.”
- “Learned how engagement happens with very little levels of investment.”
- Building the work into the system and working with government counter parts (whether at the local or national level) to ensure they are engaged and own the structure/work
- “Supporting local leadership, giving them resources. Leadership means that there is a local structure that defines what needs to be done with a local improvement team.. accountable for what is to be accomplished in a given time.”
- “…The focus on the improvement… so we can engage not only “experts” but others that can play a role in quality improvement.”

Partners

- “…. Integrating work within the existing system, rather than working linearly… working towards the same vision.”
- “Work now is really working through partners” (In India)
- “Work in Palestine required the work of partners and one huge, capable partner.”
- “partners are aware beyond the team and are working together with the team, maintaining communication to facilitate shared learning and creating tools that allow teams at the local level.”

Leadership

- “Technical capability is not enough; leadership is needed”
- “Once you have a system, then you have a leadership structure involved that is over and above the individual facilities. This requires a different level of engagement, communication.”

Structures (teams at facility level, coaches, executive structure)

Systems (HR, data systems, communication systems, management systems)

Culture (management structures, teams, individuals, problem solving) –

leadership needs to put this in place

There is failure when this management fails…
