# Supplementary material for: Managing hundreds of improvement teams
Source: F1000Res. 2018 Oct 31;7:1722. [Version 1] doi: 10.12688/f1000research.16099.1 (PMC6305208; doi:10.12688/f1000research.16099.1)
Supplement: Supplementary file 2 [file f1000research-7-17579-s0001.tgz › 20b39af9-8e56-433b-9beb-886de8b95698_Supplementary_File_1.docx]

# Supplementary File 1

## ASSIST country examples of managing hundreds of improvement teams

### India

The USAID ASSIST Project began work in India in August 2013 in six USAID-supported states to build improvement capability by enhancing the commitment and capability of leaders and healthcare workers to implement healthcare improvement along the continuum of reproductive, maternal, neonatal, child, and adolescent health (RMNCH+A). This activity resulted in a 15% reduction in perinatal mortality in the sites we supported. By the end of December 2015, the project was working with 415 facilities and 437 quality improvement (QI) teams in the country. At that point, ASSIST handed over the support of these facilities to IPE Global, another USAID-funded partner. Since February 2016, ASSIST has focused on building relationships with domestic institutions to support them to become leaders in implementing and spreading QI approaches.

|  | **India** |
| --- | --- |
| **Objective(s)** | To build improvement capability by enhancing the commitment and capability of leaders and healthcare workers to implement healthcare improvement along the continuum of reproductive, maternal, neonatal, child, and adolescent health (RMNCH+A). |
| **Scale** | Six USAID-supported states |
| **Number of Teams** | 415 facilities and 437 teams by December 2015 |
| **Time** | August 2013 – December 2015 (handover in December 2015)  February 2016 – December 2017 |
| **Results** | At the end of December 2015 ASSIST handed over the support of facilities to IPE Global, another USAID-funded partner. Since February 2016 ASSIST has focused on building relationships with domestic institutions to support them to become leaders in implementing and spreading QI approaches. |

### Tanzania

In Tanzania, the USAID ASSIST Project aimed to continue work started during the USAID Health Care Improvement Project (HCI) in 2007-2012. The main interventions included adapting and sustaining evidence-based practices to improve access to HIV testing, antiretroviral therapy (ART) enrollment, retention in care for HIV-infected patients, and ensuring wellbeing of people living with HIV (PLHIV). This was achieved through capacity building of Regional Health Management Teams (RHMTs), Council Health Management Teams (CHMTs), and continuous quality improvement (CQI) teams in analyzing and redesigning processes of care, devising new care delivery models, developing QI tools, and sharing platforms for Ministry of Health, Community Development, Gender, Elderly and Children (MOHCDGEC) structures. The initial work started with two regions and over time, additional regions were gradually added until almost the whole country was covered.

Improvement efforts focused on implementation of a package of core interventions that included scaling up ART, prevention of mother-to-child transmission of HIV (PMTCT), care and support for orphans and vulnerable children (OVC), and voluntary medical male circumcision (VMMC) services, as well as community home-based care (CHBC) volunteers in line with PEPFAR 3.0 guidance. The project also supported the development of interventions and strategies to improve the quality of HIV rapid testing as well as community-based interventions to improve linkages, ART adherence, and retention. In 2017, ASSIST supported the MOHCDGEC to build on the gains of the previous years as the country adopted the Test and Treat ART delivery model as a strategy to achieve the 90-90-90 targets.


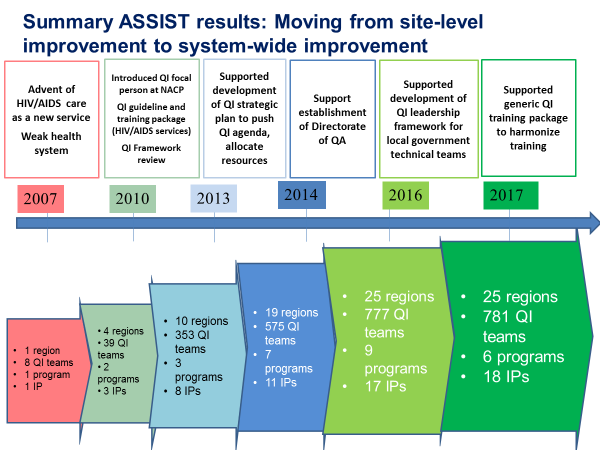


|  | **Tanzania** |
| --- | --- |
| **Objective(s)** | 1. Strengthen the capacity of the MOHCDGEC leadership and middle-level managers to continuously improve the quality of PMTCT care and lead scaling up and spread of PMTCT best practices to all implementing partners and scale-up councils  2. Support the MOHCDGEC leadership and middle-level managers to scale up improvement activities for ART services to achieve sustainable coverage, retention, and improved viral suppression  3. Support the MOHCDGEC, IPs, stakeholders, and local structures to strengthen quality of care, support, and protection to the most vulnerable children and adolescents through improvement approaches  4. Support the MOHCDGEC, local government authorities, and community-based IPs to strengthen community-based mechanisms for improving HIV testing, ART pick-up, adherence, retention as well as facility-community referrals and linkages for PLHIV  5. Work with MOHCDGEC and IPs to improve safety, increase efficiency, and the level of integration of VMMC and early infant male circumcision services into primary health services  6. Work with MOHCDGEC and IPs to improve the quality of HIV rapid testing services |
| **Scale** | 84 PEPFAR scale-up and 98 sustained councils |
| **Number of Teams** | 260 communities, 618 facilities, and 781 teams by June 2017 |
| **Time** | 2008 (under USAID HCI) – July 2017 |
| **Results** | In FY17, ASSIST provided technical support to MOHCDGEC structures, regional IPs, faith-based organizations, and community groups to apply QI methods towards attaining PEPFAR 3.0 and UNAIDS 90-90-90 goals on achieving HIV epidemic control. Learning sessions, quarterly coaching and mentoring visits, and on-the-job trainings were provided to improve the full range of HIV services in the 84 PEPFAR scale-up and 98 sustained councils, reaching a total of 618 facilities. |

### Uganda

In the last 18 years, ASSIST and predecessor projects have made significant contributions to improving patient outcomes at the facility level in Uganda in the technical areas of HIV; tuberculosis (TB); maternal, newborn, and child health (MNCH); malaria; and services for orphans and vulnerable children and their families. ASSIST has also contributed to new learning in these areas in the country, both at national and sub-national levels.

|  | **Uganda** |
| --- | --- |
| **Objective(s)** | 1. Contribute to achieving UNAIDS 90-90-90 targets  2. Keep mother-to-child transmission of HIV (MTCT) below 5% in areas where ASSIST works  3. Prevent child and maternal deaths through improving MNCH services  4. Strengthen health systems through above-site support to MOH and other IPs  5. Generate new knowledge to improve service delivery and achieve better health outcomes  6. Reduce new HIV infections among adolescent girls and young women (AGYW) and key and priority populations |
| **Scale** | 13 out of 13 regions and 65/112 districts in June 2017 |
| **Number of Teams** | 634 communities, 280 facilities, and 928 teams in June 2017 |
| **Time** | 2005 – Present  Began work in Northern Uganda in 2015 |
| **Results** | Absenteeism in six districts was reduced from 47% to 7%. Stock-outs were reduced from 54.5% to 0% in Kaliro District. Finding HIV-positive males increased by 18% among fisher folks and by over 50% among male sexual partners of female index clients. 33% reduction in the fresh stillbirth rate. 24% reduction in newborn death rate (0-7days), and 17% reduction in total perinatal death rates.  Dispensing practices, clinical wellness, and adherence to ART improved by 45%, 28%, and 20%, respectively, from baseline to end line in 14 facilities in Eastern Uganda. Reduced unjustified antibiotic prescription for cough or cold, malaria, and diarrhea by 77%, 34%, and 38%, respectively, in 10 facilities in Northern Uganda. Increased the number of TB samples processed per month by 5 GeneXpert machines in Northern Uganda from 91 to 448, and the corresponding number of identified TB-positives from 19 to 76.^^[[1]](#footnote-1)^^[[2]](#footnote-2)^^[[3]](#footnote-3)^^ |

## Spread

**Illustrative spread approaches**

Natural diffusion: this is the uptake of innovations by individuals within the social system. (Everett Rogers, *Diffusion of Innovations*)

Extension agents: heavily used in the agricultural sector in the United States. This spread involves people moving from site to site sharing experiences and best practices. In healthcare, we use coaches as extension agents.

Emergency mobilization: often used by international organizations in catastrophic events. Emergency mobilization is done very quickly and at scale to reach a large number of people in the shortest time possible.

Collaborative improvement: an approach that brings together multiple teams to work on common aims for improvement to change processes of care to improve outcomes.

Virtual collaborative: this type of collaborative is conducted through the internet. Collaboratives that require systematic changes and more collaboration need in-person interaction.

Campaign spread: an all-at-once strategy. This is an effective means of spread. For example, for immunizations.

Wave sequence spread: a type of spread that uses agents from the original sites to spread better care delivery to other sites within the system.

Hybrid models: this combines more than one element in the above-mentioned strategies. For example, extension agents may be used in collaboratives.

**Key lessons learned in spread**

Results from initial sites are key drivers for spread. Early successes and people within the system taking ownership and pride in these successes builds champions, will, and motivation; these are critical for spread.

Improvement is about change. Therefore, we must enable people to make changes in their work through:

(a) Equipping people with the ability to make changes using a change model such as the Plan-Do-Study-Act (PDSA) cycle

b) Providing assistance to teams in the form of coaching

(c) Systems thinking

(d) Enabling managers to lead spread in their systems

(e) Role modeling: the effect that the change agents can have on the members of the social system

(f) Providing normative and regulatory support

People working under constraints can be creative.

Scale-up efforts require meticulous attention to detail. Logistics and organization are key to success in scale-up. In particular, it is critical to identify resources which, while not rate-limiting during piloting, will become so during scale-up, and start planning and testing early on how to overcome these barriers.

The champions who developed the prototype are critical for leading the scale-up in the wave sequence approach.

Leadership must come from within the system.

1. Karamagi, E., Nturo, J., Donggo, P., Kyobutungi, I., Aloyo, J., Sensalire, S., & Rahimzai, M. (2017). Using quality improvement to improve the utilisation of GeneXpert testing at five lab hubs in Northern Uganda. *BMJ Open Qual*, 6(2), e000201. [↑](#footnote-ref-1)
2. Byabagambi, J. B., Broughton, E., Heltebeitel, S., Wuliji, T., & Karamagi, E. (2017). Assessment of a quality improvement intervention to strengthen pharmaceutical human resources and improve availability and use of HIV medicines in Uganda. *BMJ Open Qual*, 6(2), e000194. [↑](#footnote-ref-2)
3. Chitashvili, T., Cherkezishvili, E., Karamagi, E., & Mwanja, N. Improving rational antibiotic treatment of common childhood conditions in Uganda. *AMR Control 2017*, 75-81. [↑](#footnote-ref-3)
