# Supplementary material for: Managing hundreds of improvement teams
Source: F1000Res. 2018 Oct 31;7:1722. [Version 1] doi: 10.12688/f1000research.16099.1 (PMC6305208; doi:10.12688/f1000research.16099.1)
Supplement: Peer review history

# Peer review: Referee response for version 1

**DOI:** 10.5256/f1000research.17579.r41222

**Published:** 2018-12-14

**Authors:** David Galler

**Affiliations:** 1 Department of Intensive Care Medicine, Middlemore Hospital, Otahuhu, New Zealand

Thanks for the opportunity to read this paper which I found fascinating for a number of reasons:

1. Outlining the breadth and depth of the US Assist program.
2. The complexity of the issues addressed and the methodology of distilling down messages from the many different conversations necessary to deliver the final conclusions.
3. Making sense of really complex pieces of work to assist others in being successful is a terrific goal and I appreciate the hard work that has gone into this.

I would be interested to know given the experience and expertise of the authors whether they were in anyway surprised by the results of those conversations and whether any element of pre-test bias may have influenced the distillation process to deliver the six key components and how that was managed - they seem so intuitively correct. This is always a hard question but I think the process you used may well have overcome that inherent bias if it existed.

- Were any thoughts given to these key components in the setup of the original work? 
- The six key components and the descriptions of each of them as they relate to the Quality Management Triangle is neat and may well be of great practical support to those that will subsequently use it.
- As a next step I would like to see this framework tested from the get-go in the establishment of new work.

I have read this submission. I believe that I have an appropriate level of expertise to confirm that it is of an acceptable scientific standard.

# Peer review: Referee response for version 1

**DOI:** 10.5256/f1000research.17579.r40061

**Published:** 2018-11-16

**Authors:** Peter Lachman

**Affiliations:** 1 International Society for Quality in Health Care (ISQua) , Dublin, Ireland

This is an interesting report on the management of quality improvement teams by a large donor aid organisation. The focus is on what it takes to set up a successful programme at scale. This is one of the logistical issues to be addressed in any programme. The construct that is offered could be adapted at any level - it could be scaled down as well as scaled up.

 The framework is useful, and the authors provide a good rationale and explanation of the way to set up the programme. This would need to be tested. The question of successful implementation of projects over time is a reference to generalizability. 

 It would be interesting if this is now being tested in a prospective way. Much of what is concluded is not essentially new; what is new is the theory in the context of spread and scale up.

 A few questions need to be answered going forward:

1. Do they now use this framework when they start a new programme? 
2. Do they use the framework as a continual real time assessment of the implementation of a programme, or is it a theoretical construct?

The paper does not provide outcome measures - how do they measure the success of the framework?

1. Is there a measurement plan going forward to evaluate the framework?
2. An overview of the outcomes of their programmes would assist in demonstrating how this works. I am sure that there is a range of success and perhaps they could analyse their programmes against the framework linked to programme outcomes.

One also needs to consider the issue of sustainability. The authors state that “Improvement teams must have both the technical and leadership capability to carry out and sustain improvements to improve healthcare outcomes. To manage these improvement teams, we have developed a management structure with a ‘culture’ of quality, engagement, and empowerment”. They imply that this will result in sustainable change. It is a laudable aim, but one needs data to support the presumption of sustainability.

 One can assess this by looking at the differences between those who have followed this framework and those who have not and what happens when the programmes end. One needs to report on whether a difference in sustainability of programmes is there between those who followed this framework and those that did not to determine the outcomes long term, especially after aid and technical support is withdrawn and support has stopped. Experience in UIC is that there is a fall-off once technical support is withdrawn.

 Finally, the word ‘patient’ appears only twice in the paper. I think that it may be implied that partnerships include partnerships with patients. Unfortunately, this is not stated, and I wonder whether another dimension to consider in addition to partnerships with people who deliver and plan care, would be partnerships with patients and families in the improvement process.

 I add a recent review on spread from The Health Foundation which they could consider (Horton *et al.*, 2018 ^1^).

I have read this submission. I believe that I have an appropriate level of expertise to confirm that it is of an acceptable scientific standard.

## References

[1] HortonTIllingworthJWarburtonW: The spread challenge: How to support the successful uptake of innovations and improvements in health care. *The Health Foundation*.2018; Reference source

# Response

**Published:** 2018-11-20

**Authors:** M Rashad Massoud

**Affiliations:** Department of Public Health, University Research Co LLC, USA

Dear Peter,

Thank you very much for your thoughtful comments. We would like to respond to some of the questions you posed below, each of which are valid points.

The scope of this paper was to present the main points that resulted from discussions among senior staff on the USAID ASSIST project. Although these were recurring themes between the activities and staff working on the project, we did not develop a framework as a result of these discussions. Rather, these points are a synthesis from the discussions that show the common practices in our work. Through the group discussions and in-depth interviews described in this paper, were we able to draw out these key points and present them in this paper.

The proof of success in the use of the six interrelated categories presented in this paper is reflected in the outcomes of care that are improved in USAID ASSIST activities, as presented in the country examples in the annex of this paper. 

In response to your comment on patient-centeredness, the role of the patient is something that we apologize does not come through in this paper. In our work, it is all about the patient. This paper talks about the teams that are serving the patients and delivering care to patients. In many cases, patients are in fact part of the team, as in the case with expert patients as part of the improvement team.

Unfortunately, in the scope of this paper, we were unable to address all the points you have presented due to the interest of page limitations and conciseness. We will consider and hope to address these points in future discussions on this topic.

Thank you again for your comments.
